# Association of *CYP1A1* and microsomal epoxide hydrolase polymorphisms with lung squamous cell carcinoma

**DOI:** 10.1054/bjoc.1999.1011

**Published:** 2000-02

**Authors:** P Lin, S-L Wang, H-J Wang, K-W Chen, H-S Lee, K-J Tsai, C-Y Chen, H Lee

**Affiliations:** Institute of Toxicology and Department of Public Health, Chung-Shan Medical and Dental College, No. 110, Sec 1, Chien-Kuo N. Rd., Taichung, Taiwan, 40203, Republic of China; Department of Clinical Laboratories, Chung-Shan Memorial Hospital, Taichung, Taiwan, Republic of China; Department of Thoracic Surgery and Pathology, Veterans General Hospital-Taichung, Taiwan, Republic of China

**Keywords:** cytochrome P4501A1, microsomal epoxide hydrolase, genetic polymorphism, lung squamous cell carcinoma, Taiwan

## Abstract

Lung cancer is the leading cause of death among cancers in Taiwan. Although the etiology of lung cancer has yet to be defined, genetic variability in activities of metabolic enzymes has been correlated with lung cancer. In the present study, the possibility of association of *CYP1A1* and microsomal epoxide hydrolase (*HYL1*) genetic polymorphisms with lung cancer was examined among 132 lung cancer patients and 259 controls in Taiwan. No significant association was observed for either *CYP1A1* or *HYL1* polymorphism alone and the overall incidence of lung cancer after adjusting for age, gender and smoking status. When cases were stratified according to histological type, there was significant association between CYP1A1*2A homozygote and squamous cell carcinoma (SCC) (odds ratio (OR) 2.86; 95% confidence interval (CI) 1.33–6.12). Similarly, the proportion of *HYL1* genotypes corresponding to high or normal enzyme activities was higher in SCC than in controls (OR 1.96; 95% CI 1.04–3.70). A combination of susceptible *CYP1A1* and *HYL1* genotypes was found to be highly associated with lung cancer, especially with SCC (OR 6.76; 95% CI 2.29–19.10). Our results suggest that the combination of *CYP1A1* and *HYL1* polymorphisms is an important risk factor for lung SCC. © 2000 Cancer Research Campaign


*British Journal of Cancer* (2000) **82**(4), 852–857
© 2000 Cancer Research Campaign
DOI: 10.1054/ bjoc.1999.1011, available online at http://www.idealibrary.com on IDEAL®

### Association of *CYP1A1* and microsomal epoxide hydrolase polymorphisms with lung squamous cell carcinoma

**P Lin[1], S-L Wang[2], H-J Wang[1], K-W Chen[1], H-S Lee[2], K-J Tsai[3], C-Y Chen[4] and H Lee[1]**

[1]Institute of Toxicology and [2]Department of Public Health, Chung-Shan Medical and Dental College, No. 110, Sec 1, Chien-Kuo N. Rd., Taichung 40203, Taiwan, Republic of China; [3]Department of Clinical Laboratories, Chung-Shan Memorial Hospital, Taichung, Taiwan, Republic of China; [4]Department of Thoracic Surgery and Pathology, Veterans General Hospital-Taichung, Taiwan, Republic of China

**Summary** Lung cancer is the leading cause of death among cancers in Taiwan. Although the etiology of lung cancer has yet to be defined, genetic variability in activities of metabolic enzymes has been correlated with lung cancer. In the present study, the possibility of association of *CYP1A1* and microsomal epoxide hydrolase (*HYL1*) genetic polymorphisms with lung cancer was examined among 132 lung cancer patients and 259 controls in Taiwan. No significant association was observed for either *CYP1A1* or *HYL1* polymorphism alone and the overall incidence of lung cancer after adjusting for age, gender and smoking status. When cases were stratified according to histological type, there was significant association between *CYP1A1*2A* homozygote and squamous cell carcinoma (SCC) (odds ratio (OR) 2.86; 95% confidence interval (CI) 1.33–6.12). Similarly, the proportion of *HYL1* genotypes corresponding to high or normal enzyme activities was higher in SCC than in controls (OR 1.96; 95% CI 1.04–3.70). A combination of susceptible *CYP1A1* and *HYL1* genotypes was found to be highly associated with lung cancer, especially with SCC (OR 6.76; 95% CI 2.29–19.10). Our results suggest that the combination of *CYP1A1* and *HYL1* polymorphisms is an important risk factor for lung SCC. © 2000 Cancer Research Campaign

**Keywords**: cytochrome P4501A1; microsomal epoxide hydrolase; genetic polymorphism; lung squamous cell carcinoma; Taiwan

Cancer is the leading cause of death in Taiwan, and lung cancer is the leading and the second-leading cause of cancer deaths among women and men, respectively, in Taiwan (Department of Health, 1996). Genetic variability in metabolic activation or detoxification of environmental carcinogens partially explains host susceptibility to chemical-induced cancers (Daly et al, 1994). Carcinogens have been identified in cigarette smoke (Witschi et al, 1997), and cigarette smoking is thus strongly associated with the risk of lung squamous cell carcinoma (SCC) (Gazdar and Minna, 1997). High levels of polycyclic aromatic hydrocarbons (PAHs), such as benzo[*a*]pyrene (BaP) benzo[*g,h,i*]perylene and benzo[*b*]fluoranthene, were detected in cigarette smoke (Witschi et al, 1997) and airborne particulates collected from Taiwan (Kuo et al, 1998). PAHs are converted into dihydrodiol epoxides by cytochrome P450 enzymes and epoxide hydrolase in mammalian tissues (Hughes and Phillips, 1993; Mass et al, 1996; Josephy, 1997). These dihydrodiol epoxide metabolites attack DNA and form DNA adducts. As a consequence of DNA adduct formation, gene mutation and the initiation of carcinogenesis occurrs (Hall and Grover, 1990). DNA adducts have been detected in humans exposed to cigarette smoke and other environmental pollutants (Gallagher et al, 1993; Binkova et al, 1995).

Cytochrome P4501A1 (CYP1A1) metabolizes a range of PAHs, including BaP, and is induced by various PAHs through transcriptional activation (Denison and Whitlock, 1995). Genetic differences in human CYP1A1 enzyme inducibility have been demonstrated (Kellermann et al, 1973). Previous epidemiological studies have shown that the highly inducible phenotype of *CYP1A1* is more commonly found in lung cancer patients than in control subjects (Kouri et al, 1982). An *Msp*I polymorphic site at the 3′-flanking region of *CYP1A1* gene was identified (Kawajiri et al, 1990) and this allele was called *CYP1A1*2A* (Cascorbi et al, 1996). It has been reported that there is an association between the *CYP1A1*2A* allele and lung cancer in Japanese, white, Hawaiian and Korean populations (Kawajiri et al, 1990; Xu et al, 1996; Hong et al, 1998; Le Marchand et al, 1998). However, in other studies, using populations such as Finnish, Norwegian, German and North American, the relationship between the *CYP1A1*2A* allele and lung cancer was not observed (Tefre et al, 1991; Hirvonen et al, 1992; Shields et al, 1993; Drakoulis et al, 1994).

Microsomal epoxide hydrolase (HYL1) catalyses the hydrolysis of reactive aliphatic and arene epoxides generated by cytochrome P450 enzymes to more water-soluble dihydrodiol derivatives (Oesch, 1973). This reaction is generally considered a detoxification reaction (Oesch, 1973). In certain instances, such as PAHs, the reaction products of HYL1 may be further derivatized to secondary epoxide species by cytochrome P450 enzymes (Josephy, 1997). However, these dihydrolepoxides are often poor substrates for HYL1 and are highly reactive, attacking cellular DNA (Lu and Miwa, 1980). Epidemiological studies show that HYL1 activity greatly varies between different individuals (Omiecinski et al, 1993). Two genetic polymorphic sites of *HYL1* have been identified (Hassett et al, 1994). A T to C mutation in exon-3 of *HYL1* gene changes tyrosine residue 113 to histidine, which reduces enzyme activity at least 60% (low allele). This allele is defined as *HYL1*2*. In addition, an A to G mutation in

*Received 17 November 1998*
*Revised 10 August 1999*
*Accepted 21 September 1999*

*Correspondence to:* H Lee exon 4 changes histidine residue 139 to arginine, which increases enzyme activity at least 25% (high allele). This allele is defined as *HYL1*3*. The level of HYL1 activity may be predicted by *HYL1* genotyping (Hassett et al, 1994). *HYL1* genotypes with low enzyme activity were found to be associated with high susceptibility to chronic obstructive pulmonary disease and emphysema (Smith and Harrison, 1997). Recently, a significant association of high predicted HYL1 activity and lung cancer was found among French White smokers (Benhamou et al, 1998) Furthermore, the *HYL1*3* allele was associated with an increased risk of lung cancer in a Chinese population (Persson et al, 1999).

In the present study, we examined the distributions of genetic polymorphisms of *CYP1A1* and *HYL1* among control and lung cancer groups in Taiwan. Since CYP1A1 and HYL1 participate in bioactivation of PAHs, which are found in cigarette smoke and environmental pollutants, we investigated whether these genetic polymorphisms play a role in individual susceptibility to lung cancer in Taiwan. Furthermore, the possibility of an interaction between *CYP1A1* and *HYL1* genetic polymorphisms in association with lung cancer risk was also studied.

### MATERIALS AND METHODS

### Study population

Controls were non-cancer patients who visited cardiology, urology, or endocrinology clinics or who attended for health status examination, at Chung-Shan Memorial Hospital in Taichung from November 1997 to January 1998. Chung-Shan Memorial Hospital is a district hospital centrally located in Taichung city and most patients are residents of Taichung. In order for the age and gender distributions of controls to match those of lung cancer patients, most controls were over 40 and more than 60% of controls were male. Controls were interviewed and asked about histories of cancer, occupation and smoking status. Only individuals without history of cancer were eligible to participate as controls. DNA of control group was isolated from the whole blood using a DNA isolation kit (Qiagen, Hilden, Germany). Normal pulmonary surgical resection was performed in lung cancer patients by the Department of Thoracic Surgery at Veterans General Hospital-Taichung from 1995 to 1997. Veterans General Hospital-Taichung is a medical centre located in Taichung city and attracts people with severe diseases such as cancers. Patients, who attended Thoracic Surgery Clinic at Veterans General Hospital-Taichung from 1995 to 1997, were interviewed and asked about occupation and smoking status. All controls and cases were citizens of Taiwan. DNA from the case group was isolated from normal tissues using proteinase K digestion following phenol and chloroform extraction. We collected a total of 276 controls and 169 cases for this study. Subjects with no data on smoking status were excluded from the study, leaving 267 controls and 147 cases. Among 267 controls and 147 cases, eight control subjects and 15 cases had quit smoking. These ex-smokers were excluded from this study, leaving 259 controls and 132 cases. The histologies of lung cancer tumour types were determined according to the WHO classification method. Adenocarcinomas and squamous cell carcinomas (SCCs) were the major histological types among lung cancer patients. Of eight cases involving other histological types, three were small-cell carcinoma, two were large-cell carcinoma, one was adenosquamous cell carcinoma, one was mixed adenocarcinoma and small-cell carcinoma, and one was mixed adenocarcinoma and large-cell carcinoma.

### Genotyping of *CYP1A1*2A* polymorphism

Genotyping of *CYP1A1*2A* polymorphism (located at the 264th base downstream from the polyadenylation signal) was performed by polymerase chain reaction (PCR) amplification procedure using the primer set of 5′-TAGGAGTCTTGTCTCATGCCT-3′ and 5′-CAGTGAAGAGGTGTAGCCGCT-3′ (Hayashi et al, 1991). The amplified products were digested with *Msp*I and analysed by electrophoresis on a 2.0% agarose gel. The wild-type allele, *CYP1A1*1*, had no *Msp*I site at the 3′-end and was characterized by a 340 base pair (bp) fragment on gel. The mutant allele, *CYP1A1*2A*, carried a base substitution of thymidine to cytosine to form an *Msp*I site and was characterized by 140 and 200 bp fragments on gel. The heterozygous genotype, *CYP1A1*1/*2A*, had both alleles and was characterized by 140, 200 and 340 bp fragments.

### Genotyping of *HYL1* polymorphism

Two polymorphic sites on *HYL1* located at exon 3 and exon 4 were defined as *HYL1*2* and *HYL1*3* respectively (Smith and Harrison, 1997). Genotyping of *HYL1*2* polymorphism was performed using PCR amplification with the primer set of 5′-GATCGATAAGTTCCGTTTCACC-3′ and 5′-ATCCTTAGTCTTGAAGTGAGGAT-3′, and genotyping of *HYL1*3* polymorphism was performed with the primer set of 5′-ACATCCACTTCATCCACGT-3′ and 5′-ATGCCTCTGAGAAGCCAT-3′ (Smith and Harrison, 1997). The reaction mixture contained 120 ng of DNA, 0.2 mM of each of the deoxynucleotide triphosphates, 2 mM magnesium chloride ($MgCl_2$), 0.2 μM primer and 0.6 units of *Taq* DNA polymerase in a volume of 30 μl. The annealing temperatures for exon 3 and exon 4 PCR reaction were 51 and 59°C respectively. The amplified 162 base pair (bp) of exon 3 PCR products were digested with *Eco*RV, and the amplified 210 bp exon 4 PCR products were digested with *Rsa*I. All reactions were analysed on a 4% mixed agarose gel (agarose:low melting point agarose = 3:1). The wild-type allele on exon 3, *HYL1*1*, had an *Eco*RV site and was characterized by 140 bp and 22 bp fragments on gel. The mutant allele on exon 3, *HYL1*2*, carried a T–C substitution resulting in the loss of an *Eco*RV site, and was identified by a 162 bp fragment on gel. The wild-type allele on exon 4, *HYL1*1*, had no *Rsa*I site, but a 210 bp fragment was observed. The mutant allele in exon 4, *HYL1*3*, carried an A–G substitution to form an *Rsa*I site and was characterized by the presence of 164 and 46 bp fragments.

### Statistical analysis

Multivariate logistic regression analysis was performed to adjust for age, gender and smoking status and to assess the association between genotypes and lung cancer risk. For statistical analysis of *CYP1A1*, we combined subjects carrying the *CYP1A1*1* allele into one group. Male gender, smokers and *CYP1A1*2A* homozygote were defined as 1, and the others as 0. For analysis of *HYL1*, we combined the following genotypes into one group of ‘high/normal’ genotypes: wild-type exon 3 genotype; *HYL1*2* heterozygote with at least one *HYL1*3* allele (Smith and Harrison,

© 2000 Cancer Research Campaign

**Table 1** Characteristics of controls and lung cancer patients

| | Cases (%) | Controls (%) |
|---|---|---|
| Total | 132 | 259 |
| Gender | | |
| Male | 92 (69.7%) | 159 (61.4%) |
| Female | 40 (30.3%) | 100 (38.6%) |
| Age | | |
| Mean ± s.d. | 63 ± 9 | 58 ± 12 |
| Smoker | 75 (56.8%) | 86 (33.2%) |
| Pack-years | 39.14 ± 21.55 | 29.95 ± 25.32 |

1997). The 'low' group included *HYL1*2* homozygote; *HYL1*2* heterozygote with wild-type exon 4 genotype (Smith and Harrison, 1997). The 'high/normal' group was defined as 1. Odds ratios (ORs) were calculated to assess the relative risk with respect to genotype and were expressed together with 95% confidence interval (CI).

### RESULTS

In this study, samples were collected from 259 non-cancer controls and 132 lung cancer patients. The average age was 63 (± 9) years for the case group and 58 (± 12) years for the control group (Table 1). More than 60% of lung cancer patients and controls were male (Table 1). No significant difference in the gender distribution was observed between case and control groups (data not shown). The proportion of smokers and cumulative smoking dose (pack-years) were higher in the case group than in the control group (Table 1). When we evaluated the effects of age, gender and smoking status on the lung cancer risk by univariate logistic regression analysis, all three factors were associated with lung cancer (data not shown). To assess the association between genetic polymorphisms of *CYP1A1* and *HYL1* with lung cancer, we carried out multivariate analyses adjusted for age, gender and smoking status.

The genotypes of *CYP1A1* were determined using a PCR reaction followed by *Msp*I digestion. We combined individuals carrying the *CYP1A1*1* allele into one group to estimate the relative risk of the *CYP1A1*2A* homozygote to lung cancer. DNA of control group was isolated from blood. DNA of lung cancer group was from normal pulmonary surgical resection, because the majority of lung cancer patients had no blood sample available. In our preliminary study, we identified *CYP1A1* genotypes with DNA isolated from blood and normal pulmonary surgical resection from 17 lung cancer patients. Genotypes identified in blood DNA were exactly the same as those identified from surgical resection (data

**Table 2** Proportions of *CYP1A1*2A* homozygote among controls and lung cancer patients

| | Cases (*n* = 132) | Controls (*n* = 259) | OR (95% CI)[a] | *P*-value |
|---|---|---|---|---|
| Total | 28/132 (21.2%) | 35/259 (13.5%) | 1.60 (0.91–2.89)[b] | 0.101 |
| Gender | | | | |
| Male | 24/92 (26.1%) | 20/159 (12.6%) | 2.29 (1.12–4.66)[c] | 0.023 |
| Female | 4/40 (10.0%) | 15/100 (15.0%) | 0.64 (0.20–2.07)[c] | 0.454 |
| Smoking status | | | | |
| Smoker | 21/75 (28.0%) | 10/86 (11.6%) | 3.23 (1.29–8.06)[d] | 0.012 |
| Non-smoker | 7/57 (12.3%) | 25/173 (14.5%) | 0.82 (0.33–2.02)[d] | 0.665 |
| Histology | | | | |
| Adenocarcinoma | 10/69 (14.5%) | | 1.08 (0.50–2.32)[b] | 0.849 |
| Squamous | 16/55 (29.1%) | | 2.86 (1.33–6.12)[b] | 0.007 |
| Others | 2/8 (25.0%) | | | |

[a]Odds ratios were estimated to calculate the association between *CYP1A1*2A* homozygote and lung cancer risk. [b]Adjusted for age, gender and smoking status. [c]Adjusted for age and smoking status. [d]Adjusted for age and gender.

**Table 3.** Proportions of high/normal *HYL1* genotypes among controls and lung cancer patients

| | Cases (*n* = 132) | Controls (*n* = 259) | OR (95% CI)[a] | *P*-value |
|---|---|---|---|---|
| Total | 54/132 (40.9%) | 101/259 (39.0%) | 0.97 (0.62–1.52)[b] | 0.896 |
| Gender | | | | |
| Male | 40/92 (43.5%) | 63/159 (39.6%) | 0.85 (0.48–1.51)[c] | 0.583 |
| Female | 14/40 (35.0%) | 38/100 (38.0%) | 1.10 (0.52–2.34)[c] | 0.808 |
| Smoking status | | | | |
| Smoker | 34/75 (45.3%) | 38/86 (41.3%) | 1.04 (0.52–2.07)[d] | 0.910 |
| Non-smoker | 20/57 (35.1%) | 63/173 (36.4%) | 0.90 (0.48–1.69)[d] | 0.734 |
| Histology | | | | |
| Adenocarcinoma | 21/69 (30.4%) | | 0.65 (0.36–1.16)[b] | 0.144 |
| Squamous | 31/55 (56.4%) | | 1.96 (1.04–3.70)[b] | 0.038 |
| Others | 2/8 (25.0%) | | | |

[a]Odds ratios were estimated to calculate the association of high/normal *HYL1* genotypes and lung cancer risk. [b]Adjusted for age, gender and smoking status. [c]Adjusted for age and smoking status. [d]Adjusted for age and gender.

 © 2000 Cancer Research Campaign

**Table 4** Analysis of the interactions between *CYPIA1* and *HYL1* genotypes on their association with lung cancer risk

| | **Categories[a] (*CYP1A1/HYL1* genotypes)** | **Number (cases/controls)** | **OR[b] (95% CI)** |
|---|---|---|---|
| Total cases | CYP1A1*1/*1+CYP1A1*1/*2A/low | 66/134 | 1.00 |
| | CYP1A1*1/*1+CYP1A1*1/*2A/high + normal | 38/90 | 0.76 (0.46–1.26) |
| | CYP1A1*2A/*2A/low | 12/24 | 0.93 (0.42–2.06) |
| | CYP1A1*2A/*2A/high + normal | 16/11 | 2.56 (1.08–6.10)[c] |
| Adenocarcinoma | CYP1A1*1/*1+CYP1A1*1/*2A/low | 43/134 | 1.00 |
| | CYP1A1*1/*1+CYP1A1*1/*2A/high + normal | 16/90 | 0.52 (0.27–0.99) |
| | CYP1A1*2A/*2A/low | 5/24 | 0.63 (0.22–1.77) |
| | CYP1A1*2A/*2A/high + normal | 5/11 | 1.375 (0.44–4.28) |
| Squamous | CYP1A1*1/*1+CYP1A1*1/*2A/low | 19/134 | 1.00 |
| | CYP1A1*1/*1+CYP1A1*1/*2A/high + normal | 20/90 | 1.58 (0.76–3.27) |
| | CYP1A1*2A/*2A/low | 5/24 | 1.74 (0.54–5.65) |
| | CYP1A1*2A/*2A/high + normal | 11/11 | 6.76 (2.29–19.10)[d] |

[a]Cases and controls were divided into four categories according to *CYP1A1* and *HYL1* genotypes. *CYP1A1*1/*1 + CYP1A1*1/*2A* represented wild-type *CYP1A1* and *CYP1A1*2A* heterozygote respectively; *CYP1A1*2A/*2A* represented *CYP1A1*2A* homozygote. [b]Individuals carrying both wild-type *CYP1A1* or *CYP1A1*2A* heterozygote and low *HYL1* genotypes were defined as the baseline group. Odds ratios of the other three categories were calculated by comparing to the baseline group and adjusting for age, gender and smoking status. [c]Statistically significant with $P < 0.05$. [d]Statistically significant with $P < 0.001$.

not shown). The proportion of the *CYP1A1*2A* homozygote was 21.2% for the case group and 13.5% for the control group (Table 2). The *CYP1A1* genotype distributions within both the control and the case group fitted Hardy–Weinberg equilibrium. No association was found between *CYP1A1* genotypes and overall lung cancer after adjustments for age, gender and smoking status (Table 2). Among males, the *CYP1A1*2A* homozygote was more common in the case group than in the control group (OR 2.29; 95% CI 1.12–4.66). We also stratified the case and control groups by smoking status. Among smokers, the proportion of the *CYP1A1*2A* homozygote in cases (28.0%) was significantly higher than in controls (11.6%) (Table 2). Among smoking lung cancer patients, 54.7% (41 of 75) had SCC, 36.0% (27 of 75) had adenocarcinoma, and 9.3% (seven of 75) had other histological types (data not shown). When lung cancer group was further stratified by histological type, there was a strong association between the *CYP1A1*2A* homozygote and SCC (OR 2.86; 95% CI 1.33–6.12; $P$ = 0.007). The proportion of the *CYP1A1*2A* homozygote among SCC cases (29.1%) was much higher than among controls (13.5%, Table 2). However, the *CYP1A1*2A* homozygote was not associated with adenocarcinoma (Table 2).

The low (*HYL1*2*) and high (*HYL1*3*) genetic polymorphisms of *HYL1* were determined among the case and control groups. The frequencies of both *HYL1*2* and *HYL1*3* genotypes in the control and case groups were in Hardy–Weinberg equilibrium. In control group, the proportion of wild-type *HYL1*2* genotypes was 28.1% and the proportion of wild-type *HYL1*3* genotypes was 72.6%. We divided individuals into high, normal and low *HYL1* phenotypes according to their genotypes of *HYL1*2* and *HYL1*3* polymorphisms as described previously (Smith and Harrison, 1997) and in Materials and Methods. The proportion of high/normal genotypes was 40.9% for the case group and 39.0% for the control group (Table 3). As shown in Table 3, no significant difference was observed in proportion of high/normal genotype between the control and case groups. When cases were stratified by histological type, the proportion of high/normal *HYL1* genotypes in the SCC group was significantly higher than in the control group (OR 1.96; 95% CI 1.04–3.70; $P$ = 0.038).

Since either the *CYP1A1*2A* homozygote or high/normal *HYL1* genotypes was associated with SCC, we further analysed the interaction between these two polymorphisms on their association with lung cancer. Case and control groups were divided into four categories according to *CYP1A1* and *HYL1* genotypes. Individuals with *CYP1A1*1* homozygote and low *HYL1* genotypes were defined as the baseline group. The ORs of the other three categories were estimated by comparison with the baseline group. As shown in Table 4, the OR for lung cancer with a combination of *CYP1A1*2A* homozygote and high/normal *HYL1* genotypes was 2.56 (95% CI 1.08–6.10). When cases were stratified by histological type, the OR for SCC was 6.76 (95% CI 2.29–19.10), a marked increase. A combination of susceptible *CYP1A1* and *HYL1* genotypes was not found to associate with adenocarcinoma (Table 4).

### DISCUSSION

Exposure to air pollutants, such as cigarette smoke, asbestos and PAHs is considered to be at least a partial causal factor in the development of lung cancers (Coultas and Samet, 1992; Hemminki and Pershagen, 1994). Many environmental carcinogens require metabolic activation by phase I enzymes, which show genetic variability in activity among individuals. This genetic variability may be responsible for the individual susceptibility to chemical carcinogenesis (Daly et al, 1994). Recently, genetic variation in phase I enzymes has been linked to the occurrence of lung cancers (Rannug et al, 1995). Here we explored the possible associations between genetic polymorphisms of phase I enzymes, including *CYP1A1* and *HYL1*, and lung cancer risk in Taiwan. We found that *CYP1A1*2A/*2A* and high/normal *HYL1* genotypes were associated with lung SCC, with an OR of 2.86 and 1.96 respectively (Tables 2 and 3). The OR for SCC with both susceptible *CYP1A1* and *HYL1* genotypes was further elevated to 6.76 (95% CI 2.29–19.10). These results indicate that the combination of *CYP1A1* and *HYL1* genetic polymorphisms is an important genetic risk factor for lung SCC in Taiwan.

Recently, mortality from lung cancers in Taiwan has been increasing (Department of Health, 1996). Cigarette smoking is

© 2000 Cancer Research Campaign closely associated with the risk of SCC (Gazdar and Minna, 1997) and cigarette smoke induces lung tumours in mice (Witschi et al, 1997). In addition, epidemiological studies indicate that lung cancer mortality rate is correlated with the level of air pollution and mutagenicity of airborne particles (Pershagen and Simonato, 1990). PAHs are important mutagens identified in cigarette smoke (Witschi et al, 1997) and in airborne particles collected from major cities in Taiwan (Kuo et al, 1998). PAHs induce CYP1A1 enzyme activity and also require metabolic activation by CYP1A1 and HYL1 (Denison and Whitlock, 1995; Josephy, 1997). For example, BaP is first metabolized by CYP1A1 to form BaP-7,8-oxide which is further hydrolysed by HYL1 to give the corresponding BaP-7,8-dihydrodiol (Josephy, 1997). This compound is then oxidized by cytochrome P450 enzymes to the ultimate carcinogen, 7,8-dihydroxy-9,10-epoxy-7,8,9,10-tetrahydro-BaP (BPDE) which directly attacks cellular DNA (Binkova et al, 1995). Recently, a correlation was found among smoking lung cancer patients between *HYL1* polymorphisms and BPDE adducts to serum albumin and DNA (Pastorelli et al, 1998). Patients carrying 'low activity' *HYL1* polymorphisms, two *HYL1*2* alleles and no *HYL1*3* allele, had a lower frequency of BPDE-serum albumin adduct and no DNA adducts ($P$ = 0.06). In the present study, individuals with *CYP1A1*2* homozygote and genotype corresponding to high HYL1 enzyme activity were more susceptible to lung cancer, especially SCC. It is plausible that individuals with these specific genotypes have high metabolic activation capability to convert PAHs into carcinogenic metabolites. Thus, our results suggest that PAHs play a role in the incidence of lung SCC in Taiwan. One previous study demonstrated a positive correlation for CYP1A1 and HYL1 enzyme activities with mortality among male lung cancer patients (Bartsch et al, 1992). It is worthwhile to analyse further the effect of *CYP1A1* and *HYL1* genotype combinations on survival rates of lung cancer patients in the future.

The association of the *CYP1A1*2A* allele with increased risk of lung SCC has been observed in Japanese, white and Hawaiian populations (Kawajiri et al, 1990; Le Marchard et al, 1998). The proportions of *CYP1A1*2A* homozygote reported in the Japanese population and our present study (10.6% and 13.5% respectively) were much higher than those reported for Whites (0.7–1.7%). Hence, *CYP1A1* polymorphism may play a more important role in Asian populations. Similarly, the relationship between *HYL1* genetic polymorphisms and lung cancer risk has been recently reported (Benhamou et al, 1998; Persson et al, 1999). Benhamou et al (1998) reported an increased risk of lung cancer among individuals with high and normal activity genotypes in French White smokers. We also observed the association of high and normal genotypes with SCC. The distributions of control subjects with predicted low, normal and high HYL1 activities were similar between our study (55.8%, 31.4% and 12.8%, data not shown) and Benhamou's study (49.4%, 37.8% and 12.8%). Therefore, it is unlikely that the ethnic factor contributes to the difference observed among smokers between the two studies. Alternatively, it is possible that smoking habits and components in cigarettes may differ between the two countries, and this may affect the relationship between *HYL1* genotypes and lung cancer risk among smokers. Therefore, the role of *HYL1* polymorphisms in smoking-associated lung cancer in Taiwan is still unclear.

The functional effect of *CYP1A1*2A* polymorphism has been studied in peripheral mitogen-treated lymphocytes from a Japanese population (Kiyohara et al, 1998). Among 108 lung cancer patients and 95 healthy control individuals, CYP1A1 inducibility (3-methylcholanthrene-induced/non-induced activity) in subjects carrying *CYP1A1*2A* homozygote was significantly higher than those of the other two genotypes (Kiyohara et al, 1998). However, no other functional studies for the *CYP1A1*2A* allele have been reported. Alternatively, it is possible that the *CYP1A1*2A* polymorphism may be closely linked to another gene and its association with lung cancer risk may not be related to the *CYP1A1* itself. More experimental data is required to support the correlation between genotype and phenotype. The biochemical mechanism of variation in HYL1 enzyme activity among specific genotypes has also been studied (Hassett et al, 1994). The variant amino acid in exon 3 or exon 4 was constructed in *HYL1* cDNA and expressed in vitro by transient transfection of COS-1 cells. The relative amounts of HYL1 proteins and enzyme activities were markedly different. It has been suggested that *HYL1* polymorphisms alter enzymatic function through modification of protein stability (Hassett et al, 1994). However, HYL1 enzyme activity/protein levels failed to correlate with *HYL1*2* and *HYL1*3* polymorphisms in human liver tissue (Hassett et al, 1997). New *HYL1* polymorphic sites identified at 5′-flanking region might also contribute to the variation in *HYL1* expression (Raaka et al, 1998). Therefore, the relationship between HYL1 enzyme activity and exon 3 and exon 4 polymorphisms is still uncertain. HYL1 enzyme activity is induced by cigarette smoke (Gielen et al, 1979). A significant increase in HYL1 activities of human lung tissues was found among smokers, as compared with non-smokers (Bartsch et al, 1992). Further studies are needed to clarify the relationship of exon 3 and exon 4 *HYL1* polymorphisms with HYL1 enzyme activity and inducibility distribution in populations.

In summary, this is the first study to demonstrate an extremely strong association of combined *CYP1A1* and *HYL1* genetic polymorphisms with lung cancer, especially for SCC. These results suggest that cigarette smoke and environmental pollutants contribute to the development of lung SCC in Taiwan.

### ACKNOWLEDGEMENTS

This work was supported by Grant DOH86-HR-611 from the National Health Research Institute, Department of Health, The Executive Yuan, Republic of China. We would like to thank Ya-Wen Cheng and Chao-Ping Chen for their help in preparing DNA from pulmonary surgical resections. We are also very grateful to Dr Yu-Mei Hsueh for assistance with data analysis.

### REFERENCES

Bartsch H, Petruzzelli S, De Flora S, Hietanen E, Camus A-M, Castegnaro M, Alexandrov K, Rojas M, Saracci R and Giuntini C (1992) Carcinogen metabolism in human lung tissues and the effect of tobacco smoking: results from a case–control multicenter study on lung cancer patients. *Environ Health Perspect* **98:** 119–124

Benhamou S, Reinikainen M, Bouchardy C, Dayer P and Hirvonen A (1998) Association between lung cancer and microsomal epoxide hydrolase genotypes. *Cancer Res* **58:** 5291–5293

Binkova B, Lewtas J, Miskova I, Lenicek J and Sram R (1995) DNA adducts and personal air monitoring of carcinogenic polycyclic aromatic hydrocarbons in an environmentally exposed population. *Carcinogenesis* **16:** 1037–1046

Cascorbi I, Brockmoller J and Roots I (1996) A C4887A polymophism in exon 7 of human CYP1A1: population frequency, mutation linkages, and impact lung cancer susceptibility. *Cancer Res* **56:** 4965–4969

Coultas DB and Samet JM (1992) Occupational lung cancer. *Clin Chest Med* **13:** 341–354

 © 2000 Cancer Research Campaign

Daly AK, Cholerton S, Armstrong M and Idle J R (1994) Genotyping for polymorphisms in xenobiotic metabolism as a predictor of disease susceptibility. *Environ Health Perspect* **102** (Suppl. 9): 55–61

Denison MS and Whitlock JP Jr (1995) Xenobiotic-inducible transcription of cytochrome P450 genes. *J Biol Chem* **270**: 18175–18178

Department of Health (1996) Republic of China: General Health Statistics. In: *Health and Vital Statistics*, pp. 88–111. Republic of China Press: Taipei

Drakoulis N, Cascorbi I, Brockmoller J, Gross CR and Roots I (1994) Polymorphisms in the human CYP1A1 gene as susceptibility factors for lung cancer. *Clin Invest* **72**: 240–248

Gallagher J, Mumford J, Li X, Shank T, Manchester D and Lewtas J (1993) *DNA Adduct Profiles and Levels in Placenta, Blood and Lung in Relation to Cigarette Smoking and Smoky Coal Emissions*, pp. 283–292. IARC Scientific Publication no. **124**: IARC: Lyon

Gazdar AF and Minna JD (1997) Cigarettes, sex, and lung adenocarcinoma. *J Natl Cancer Inst* **89**: 1563–1565.

Gielen JE, Goujon F, Sele J and Van Canfort J (1979) Organ specificity of induction of activating and inactivating enzymes by cigarette smoke and cigarette smoke condensate. *Arch Toxicol* **2**(Suppl): 239–251

Hall M and Grover PL (1990) Polycyclic aromatic hydrocarbons: metabolism, activation, and tumor initiation. In: *Chemical Carcinogenesis and Mutagenesis I*. Cooper CS and Grover PL (eds), pp 327–372. Springer-Verlag: Berlin

Hassett C, Aicher L, Sidhu JS and Omiecinski CJ (1994) Human microsomal epoxide hydrolase: genetic polymorphism and functional expression in vitro of amino acid variants. *Hum Mol Genet* **3**: 421–428

Hassett C, Lin J, Carty CL, Laurenzana EM and Omiecinski CJ (1997) Human hepatic microsomal epoxide hydrolase: comparative analysis of polymorphic expression. *Arch Biochem Biophys* **337**: 275–283

Hayashi S, Watanabe J, Nakachi K and Kawajiri K (1991) Genetic linkage of lung cancer-associated *Msp*I polymorphisms with amino acid replacement in the heme binding region of the human cytochrome P4501A1 gene. *J Biochem* **110**: 407–411

Hemminki K and Pershagen G (1994) Cancer risk of air pollution: epidemiological evidence. *Environ Health Perspect* **10**(Suppl 4): 187–192

Hirvonen A, Husgafvel-Pursiainen K, Karjalainen A, Anttila S and Vainio H (1992) Point-mutational *Msp*I and Ile/Val polymorphisms closely linked in the CYP1A1 gene: lack of association with susceptibility to lung cancer in a Finnish study population. *Cancer Epidemiol Biomark Prevent* **1**: 485–489

Hong YS, Chang JH, Kwon OJ, Ham YA and Choi JH (1998) Polymorphism of the CYP1A1 and glutathione-S-transferase gene in Korean lung cancer patients. *Exp Mol Med* **30**: 192–198

Hughes NC and Phillips DH (1993) $^{32}$P-postlabelling analysis of the covalent binding of benzo[*ghi*]perylene to DNA in vivo and in vitro. *Carcinogenesis* **14**: 127–133

Josephy PD (1997) Polycyclic aromatic hydrocarbon carcinogenesis. In: *Molecular Toxicology*, Josephy PD (ed), pp. 338–344. Oxford University Press: New York

Kawajiri K, Nakachi K, Imai K, Yoshii A, Shinoda N and Watanabe J (1990) Identification of genetically high risk individuals to lung cancer by DNA polymorphisms of the cytochrome P450IA1 gene. *FEBS Lett* **263**: 131–133

Kawajiri K, Nakachi K, Imai K, Watanabe J and Hayashi S (1993) The CYP1A1 gene and cancer susceptibility. *Crit Rev Oncol Hematol* **14**: 77–87.

Kellermann G, Shaw CR and Luyten-Kellerman M (1973) Aryl hydrocarbon hydroxylase inducibility and bronchogenic carcinoma. *N Engl J Med* **289**: 934–937

Kiyohara C, Hirohata T and Inutsuka S (1996) The relationship between aryl hydrocarbon hydroxylase and polymorphisms of the CYP1A1 gene. *Jpn J Cancer Res* **87**: 18–24

Kiyohara C, Nakanishi Y, Inutsuka S, Takayama K, Hara N, Motohiro A, Tanaka K, Kono S and Hirohata T (1998) The relationship between CYP1A1 aryl hydrocarbon hydroxylase activity and lung cancer in a Japanese population. *Pharmacogenetics* **8**: 315–523

Kouri RE, McKinney CE, Slomiany DJ, Snodgrass DR, Wray NP and McLemore TL (1982) Positive correlation between high aryl hydrocarbon hydroxylase activity and primary lung cancer as analyzed in cryopreserved lymphocytes. *Cancer Res* **42**: 5030–5037

Kuo C-Y, Chen C-Y, Cheng Y-W and Lee H (1998) Correlation between the amounts of polycyclic aromatic hydrocarbons and mutagenicity of airborne particulate samples from Taichung city, Taiwan. *Environ Res* **78**: 43–49

Le Marchand L, Sivaraman L, Pierce L, Seifried A, Lum A, Wilkens LR and Lau AF (1998) Association of CYP1A1, GSTM1, and CYP2E1 polymorphisms with lung cancer suggest cell type specificities to tobacco carcinogens. *Cancer Res* **58**: 4858–4863

Lu AY and Miwa GT (1980) Molecular properties and biological functions of microsomal epoxide hydrase. *Ann Rev Pharmacol Toxicol* **20**: 513–531

Mass MJ, Abu-Shakra A, Roop BC, Nelson G, Galati AJ, Stoner GD, Nesnow S and Ross JA (1996) Benzo [*b*]fluoranthene: tumorigenicity in strain A/J mouse lungs, DNA adducts and mutations in the Ki-*ras* oncogene. *Carcinogenesis* **17**: 1701–1704

Oesch F (1973) Mammalian epoxide hydrase: inducible enzymes catalysing the inactivation of carcinogenic and cytotoxic metabolites derived from aromatic and olefinic compounds. *Xenobiotica* **3**: 305–340

Omiecinski CJ, Aicher L, Holubkov R and Checkoway H (1993) Human peripheral lymphocytes as indicators of microsomal epoxide hydrolase activity in liver and lung. *Pharmacogenetics* **3**: 150–158

Pastorelli R, Guanci M, Cerri A, Negri E, La Vecchia C, Fumagalli F, Mezzetti M, Cappelli R, Panigalli T, Fanelli R and Airoldi L (1998) Impact of inherited polymorphisms in glutathione s-transferease M1, microsomal epoxide hydrolase, cytochrome P450 enzymes on DNA, and blood protein adducts of benzo[*a*]pyrene-diolepoxide. *Cancer Epidemiol Biomark Prevent* **7**: 703–709

Pershagen G and Simonato L (1990) Epidemiological evidence on air pollution and cancer. In: *Air Pollution and Human Cancer*, Tomatis L (ed), pp 63–74. Springer-Verlag: Heidelberg

Persson I, Johansson I, Lou Y-C, Yue Q-Y, Duan L-S, Bertilsson L and Ingelman-Sundberg M (1999) Genetic polymorphism of xenobiotic metabolizing enzymes among Chinese lung cancer patients. *Int J Cancer* **81**: 325–329

Raaka S, Hassett C and Omiecinski CJ (1998) Human microsomal epoxide hydrolase: 5′-flanking region genetic polymorphisms. *Carcinogenesis* **19**: 387–393

Rannug A, Alexandrie A, Persson I and Ingelman-Sundberg M (1995) Genetic polymorphism of cytochromes P4501A1, 2D6 and 2E1: regulation and toxicological significance. *J Occu Environ Med* **37**: 25–36

Shields PG, Bowman EG and Harrington AM (1993) Polycyclic aromatic hydrocarbon DNA adducts in human lung and cancer susceptibility genes. *Cancer Res* **53**: 3486–3492

Smith CAD and Harrison DJ (1997) Association between polymorphism in gene for microsomal epoxide hydrolase and susceptibility to emphysema. *Lancet* **350**: 630–633

Tefre T, Ryberg D, Haugen A, Nebert DW, Skaug V, Brogger A and Borresen AL (1991) Human CYP1A1 (cytochrome P1450) gene: lack of association between the *Msp*I restriction fragment length polymorphism and incidence of lung cancer in a Norwegian population. *Pharmacogenetics* **1**: 20–25

Witschi H, Espiritu I, Maronpot RR, Pinkerton KE and Jones AD (1997) The carcinogenic potential of the gas phase of environmental tobacco smoke. *Carcinogenesis* **18**: 2035–2042

Xu X, Kelsey KT, Wiencke JK, Wain JC and Christiani DC (1996) Cytochrome P450 CYP1A1 *Msp*I polymorphism and lung cancer susceptibility. *Cancer Epidemiol Biomark*. *Prevent* **5**: 687–692

*© 2000 Cancer Research Campaign*

## References

[PDF_00518] Bartsch H., Petruzzelli S., De Flora S., Hietanen E., Camus A. M., Castegnaro M., Alexandrov K., Rojas M., Saracci R., Giuntini C. (1992). Carcinogen metabolism in human lung tissues and the effect of tobacco smoking: results from a case--control multicenter study on lung cancer patients.. Environ Health Perspect.

[PDF_00523] Benhamou S., Reinikainen M., Bouchardy C., Dayer P., Hirvonen A. (1998). Association between lung cancer and microsomal epoxide hydrolase genotypes.. Cancer Res.

[PDF_00526] Binková B., Lewtas J., Misková I., Lenícek J., Srám R. (1995). DNA adducts and personal air monitoring of carcinogenic polycyclic aromatic hydrocarbons in an environmentally exposed population.. Carcinogenesis.

[PDF_00529] Cascorbi I., Brockmöller J., Roots I. (1996). A C4887A polymorphism in exon 7 of human CYP1A1: population frequency, mutation linkages, and impact on lung cancer susceptibility.. Cancer Res.

[PDF_00532] Coultas D. B., Samet J. M. (1992). Occupational lung cancer.. Clin Chest Med.

[PDF_00538] Daly A. K., Cholerton S., Armstrong M., Idle J. R. (1994). Genotyping for polymorphisms in xenobiotic metabolism as a predictor of disease susceptibility.. Environ Health Perspect.

[PDF_00541] Denison M. S., Whitlock J. P. (1995). Xenobiotic-inducible transcription of cytochrome P450 genes.. J Biol Chem.

[PDF_00545] Drakoulis N., Cascorbi I., Brockmöller J., Gross C. R., Roots I. (1994). Polymorphisms in the human CYP1A1 gene as susceptibility factors for lung cancer: exon-7 mutation (4889 A to G), and a T to C mutation in the 3'-flanking region.. Clin Investig.

[PDF_00548] Gallagher J., Mumford J., Li X., Shank T., Manchester D., Lewtas J. (1993). DNA adduct profiles and levels in placenta, blood and lung in relation to cigarette smoking and smoky coal emissions.. IARC Sci Publ.

[PDF_00552] Gazdar A. F., Minna J. D. (1997). Cigarettes, sex, and lung adenocarcinoma.. J Natl Cancer Inst.

[PDF_00554] Gielen J. E., Goujon F., Sele J., Van Canfort J. (1979). Organ specificity of induction of activating and inactivating enzymes by cigarette smoke and cigarette smoke condensate.. Arch Toxicol Suppl.

[PDF_00560] Hassett C., Aicher L., Sidhu J. S., Omiecinski C. J. (1994). Human microsomal epoxide hydrolase: genetic polymorphism and functional expression in vitro of amino acid variants.. Hum Mol Genet.

[PDF_00564] Hassett C., Lin J., Carty C. L., Laurenzana E. M., Omiecinski C. J. (1997). Human hepatic microsomal epoxide hydrolase: comparative analysis of polymorphic expression.. Arch Biochem Biophys.

[PDF_00567] Hayashi S., Watanabe J., Nakachi K., Kawajiri K. (1991). Genetic linkage of lung cancer-associated MspI polymorphisms with amino acid replacement in the heme binding region of the human cytochrome P450IA1 gene.. J Biochem.

[PDF_00571] Hemminki K., Pershagen G. (1994). Cancer risk of air pollution: epidemiological evidence.. Environ Health Perspect.

[PDF_00573] Hirvonen A., Husgafvel-Pursiainen K., Karjalainen A., Anttila S., Vainio H. (1992). Point-mutational MspI and Ile-Val polymorphisms closely linked in the CYP1A1 gene: lack of association with susceptibility to lung cancer in a Finnish study population.. Cancer Epidemiol Biomarkers Prev.

[PDF_00577] Hong Y. S., Chang J. H., Kwon O. J., Ham Y. A., Choi J. H. (1998). Polymorphism of the CYP1A1 and glutathione-S-transferase gene in Korean lung cancer patients.. Exp Mol Med.

[PDF_00580] Hughes N. C., Phillips D. H. (1993). 32P-postlabelling analysis of the covalent binding of benzo[ghi]perylene to DNA in vivo and in vitro.. Carcinogenesis.

[PDF_00590] Kawajiri K., Nakachi K., Imai K., Watanabe J., Hayashi S. (1993). The CYP1A1 gene and cancer susceptibility.. Crit Rev Oncol Hematol.

[PDF_00586] Kawajiri K., Nakachi K., Imai K., Yoshii A., Shinoda N., Watanabe J. (1990). Identification of genetically high risk individuals to lung cancer by DNA polymorphisms of the cytochrome P450IA1 gene.. FEBS Lett.

[PDF_00592] Kellermann G., Shaw C. R., Luyten-Kellerman M. (1973). Aryl hydrocarbon hydroxylase inducibility and bronchogenic carcinoma.. N Engl J Med.

[PDF_00595] Kiyohara C., Hirohata T., Inutsuka S. (1996). The relationship between aryl hydrocarbon hydroxylase and polymorphisms of the CYP1A1 gene.. Jpn J Cancer Res.

[PDF_00598] Kiyohara C., Nakanishi Y., Inutsuka S., Takayama K., Hara N., Motohiro A., Tanaka K., Kono S., Hirohata T. (1998). The relationship between CYP1A1 aryl hydrocarbon hydroxylase activity and lung cancer in a Japanese population.. Pharmacogenetics.

[PDF_00602] Kouri R. E., McKinney C. E., Slomiany D. J., Snodgrass D. R., Wray N. P., McLemore T. L. (1982). Positive correlation between high aryl hydrocarbon hydroxylase activity and primary lung cancer as analyzed in cryopreserved lymphocytes.. Cancer Res.

[PDF_00606] Kuo C. Y., Cheng Y. W., Chen C. Y., Lee H. (1998). Correlation between the amounts of polycyclic aromatic hydrocarbons and mutagenicity of airborne particulate samples from Taichung City, Taiwan.. Environ Res.

[PDF_00609] Le Marchand L., Sivaraman L., Pierce L., Seifried A., Lum A., Wilkens L. R., Lau A. F. (1998). Associations of CYP1A1, GSTM1, and CYP2E1 polymorphisms with lung cancer suggest cell type specificities to tobacco carcinogens.. Cancer Res.

[PDF_00613] Lu A. Y., Miwa G. T. (1980). Molecular properties and biological functions of microsomal epoxide hydrase.. Annu Rev Pharmacol Toxicol.

[PDF_00615] Mass M. J., Abu-Shakra A., Roop B. C., Nelson G., Galati A. J., Stoner G. D., Nesnow S., Ross J. A. (1996). Benzo[b]fluoranthene: tumorigenicity in strain A/J mouse lungs, DNA adducts and mutations in the Ki-ras oncogene.. Carcinogenesis.

[PDF_00619] Oesch F. (1973). Mammalian epoxide hydrases: inducible enzymes catalysing the inactivation of carcinogenic and cytotoxic metabolites derived from aromatic and olefinic compounds.. Xenobiotica.

[PDF_00622] Omiecinski C. J., Aicher L., Holubkov R., Checkoway H. (1993). Human peripheral lymphocytes as indicators of microsomal epoxide hydrolase activity in liver and lung.. Pharmacogenetics.

[PDF_00625] Pastorelli R., Guanci M., Cerri A., Negri E., La Vecchia C., Fumagalli F., Mezzetti M., Cappelli R., Panigalli T., Fanelli R. (1998). Impact of inherited polymorphisms in glutathione S-transferase M1, microsomal epoxide hydrolase, cytochrome P450 enzymes on DNA, and blood protein adducts of benzo(a)pyrene-diolepoxide.. Cancer Epidemiol Biomarkers Prev.

[PDF_00634] Persson I., Johansson I., Lou Y. C., Yue Q. Y., Duan L. S., Bertilsson L., Ingelman-Sundberg M. (1999). Genetic polymorphism of xenobiotic metabolizing enzymes among Chinese lung cancer patients.. Int J Cancer.

[PDF_00636] Raaka S., Hassett C., Omiencinski C. J. (1998). Human microsomal epoxide hydrolase: 5'-flanking region genetic polymorphisms.. Carcinogenesis.

[PDF_00638] Rannug A., Alexandrie A. K., Persson I., Ingelman-Sundberg M. (1995). Genetic polymorphism of cytochromes P450 1A1, 2D6 and 2E1: regulation and toxicological significance.. J Occup Environ Med.

[PDF_00641] Shields P. G., Bowman E. D., Harrington A. M., Doan V. T., Weston A. (1993). Polycyclic aromatic hydrocarbon-DNA adducts in human lung and cancer susceptibility genes.. Cancer Res.

[PDF_00644] Smith C. A., Harrison D. J. (1997). Association between polymorphism in gene for microsomal epoxide hydrolase and susceptibility to emphysema.. Lancet.

[PDF_00647] Tefre T., Ryberg D., Haugen A., Nebert D. W., Skaug V., Brøgger A., Børresen A. L. (1991). Human CYP1A1 (cytochrome P(1)450) gene: lack of association between the Msp I restriction fragment length polymorphism and incidence of lung cancer in a Norwegian population.. Pharmacogenetics.

[PDF_00651] Witschi H., Espiritu I., Maronpot R. R., Pinkerton K. E., Jones A. D. (1997). The carcinogenic potential of the gas phase of environmental tobacco smoke.. Carcinogenesis.

[PDF_00654] Xu X., Kelsey K. T., Wiencke J. K., Wain J. C., Christiani D. C. (1996). Cytochrome P450 CYP1A1 MspI polymorphism and lung cancer susceptibility.. Cancer Epidemiol Biomarkers Prev.

